# Role of Cobalt(III) Cationic Complexes in the Self-Assembling Process of a Water Soluble Porphyrin

**DOI:** 10.3390/ijms22010039

**Published:** 2020-12-22

**Authors:** Nadia Manganaro, Roberto Zagami, Mariachiara Trapani, Maria Angela Castriciano, Andrea Romeo, Luigi Monsù Scolaro

**Affiliations:** 1 Dipartimento di Scienze Chimiche, Biologiche, Farmaceutiche ed Ambientali, University of Messina and C.I.R.C.M.S.B V.le F. Stagno D’Alcontres, 31-98166 Messina, Italy; nadiamanganaro87@gmail.com (N.M.); anromeo@unime.it (A.R.); 2 CNR—ISMN Istituto per lo Studio dei Materiali Nanostrutturati c/o Dipartimento di Scienze Chimiche, Biologiche, Farmaceutiche ed Ambientali, University of Messina, V.le F. Stagno D’Alcontres, 31-98166 Messina, Italy; roberto.zagami@ismn.cnr.it (R.Z.); mariachiara.trapani@cnr.it (M.T.); maria.castriciano@cnr.it (M.A.C.)

**Keywords:** porphyrins, J-aggregates, aggregation kinetics, symmetry breaking, chiral supramolecular assemblies

## Abstract

Under moderate acidic conditions, the cationic (+3) complexes ions tris(1,10-phenanthroline)cobalt(III), [Co(*phen*)_3_]^3+^, and hexamminecobalt(III), [Co(NH_3_)_6_]^3+^, efficiently promote the self-assembling process of the diacid 5,10,15,20-*tetrakis*(4-sulfonatophenyl)porphyrin (H_2_TPPS_4_) into J-aggregates. The growth kinetics have been analyzed according to a well-established autocatalytic model, in which the rate determining step is the initial formation of a nucleus containing *m* porphyrin units (in the range 2–3), followed by a stage whose rate constant *k_c_* evolves as a power of time. The observed catalytic rate constants and the extent of J-aggregation increase on increasing the metal complex concentration, with the *phen* complex being the less active. The UV/Vis extinction spectra display quite broad envelops at the J-band, especially for the amino-complex, suggesting that electronic dipolar coupling between chromophores is operative in these species. The occurrence of spontaneous symmetry breaking has been revealed by circular dichroism and the measured dissymmetry *g*-factor decreases on increasing the aggregation rates. The role of these metal complexes on the growth and stabilization of porphyrin nano-assemblies is discussed in terms of the different degree of hydrophilicity and hydrogen bonding ability of the ligands present in the coordination sphere around the metal center.

## 1. Introduction

Aggregation is a well-known phenomenon in the field of porphyrinoids [1]. As consequence porphyrin aggregates are subject to intensive research, either for their fundamental properties, e.g., expression of chirality [2,3], or for manifold potential applications, e.g., optoelectronics or catalysis [4,5,6,7,8,9,10]. In this framework, water soluble sulfonato-porphyrins are basic building blocks to access a wide range of supramolecular self-assembled systems. Non-covalent interactions have been exploited to prepare both homo- and hetero-aggregated structures, together with mixed organic-inorganic nanosystems, whose peculiar electronic properties have received large attention in the last decades. Among the various local structural motifs, these porphyrins are able to interact adopting an edge-to-edge (J-type) or a side-by-side (H-type) geometry, each leading to specific spectroscopic features in terms of bathochromic or hypsochromic shifts in the corresponding UV/Vis electronic spectra [11]. Strong electronic coupling among the chromophores is responsible for resonant enhancement of Rayleigh [12,13,14,15,16,17,18,19,20] and Raman light scattering [21,22,23,24,25] and non-linear optical effects in large J-aggregates [26,27,28,29,30,31]. In addition, peculiar chiroptical properties emerge when these supramolecular systems grow in the presence of chemical [22,32,33,34,35,36,37,38,39,40,41,42] or physical chiral bias [43,44,45,46,47,48,49,50]. Spontaneous symmetry breaking has been also reported [51,52], making a strong speculative point for the emergence of homochirality in life.

Among the different derivatives, the tetra-anionic 5,10,15,20-*tetrakis*(4-sulfonatophenyl) porphyrin (TPPS_4_) has been one of the most studied compounds. Since the earlier report by Pasternack et al. [53] on the observation of a unusual bathochromically shifted B-band in acidified solution of the closely related TPPS_3_ porphyrin, a growing interest toward this simple symmetrical molecule and its J-aggregates has grown in literature [40,54,55,56,57,58]. Depending on the concentration of porphyrin, pH and ionic strength, J-aggregates exhibiting a quite large variety of morphologies were reported [40,59,60,61]. Despite this plethora of nano and mesoscopic structures, these species are specifically characterized by the presence of an intense and narrow band shifted to lower energy at about 490 nm (the so-called J-band) and a less intense band located at higher energy at about 420 nm (H-band). These features originate by exciton coupling of electronic transition moments according to Kasha model [11].

Different studies pointed out the important role played by hydrogen bonding [62,63,64], counter-anions [63], and specific cationic species [16,34,42,65,66,67,68,69,70,71,72,73] in governing the kinetics of growth and, consequently, the general optical and chiroptical features of these systems. Some cations, such as Na^+^ or K^+^ exert the mere role of increasing the ionic strength thus decreasing the electrostatic repulsion between adjacent porphyrins and favoring the formation of an initial dimeric aggregate [58,60]. Ni^2+^ or Zn^2+^ are effective in stabilize these species [74,75], even if some specific role in the build-up of a mesoscopic network and in the chiroptical response has been recently pointed out for this latter metal ion [65]. Some chiral complex metal cations are able to specifically interact with the growing J-aggregates imprinting their chirality in the final structures [70,71]. Spermine and similar polyamines showed the ability to drive the self-assembling process leading to fractal or sea-urchin like structures, in which a dipolar coupling mechanism has been invoked to explain the unusually broad J-bands [16,17,76]. 

**Scheme 1:**
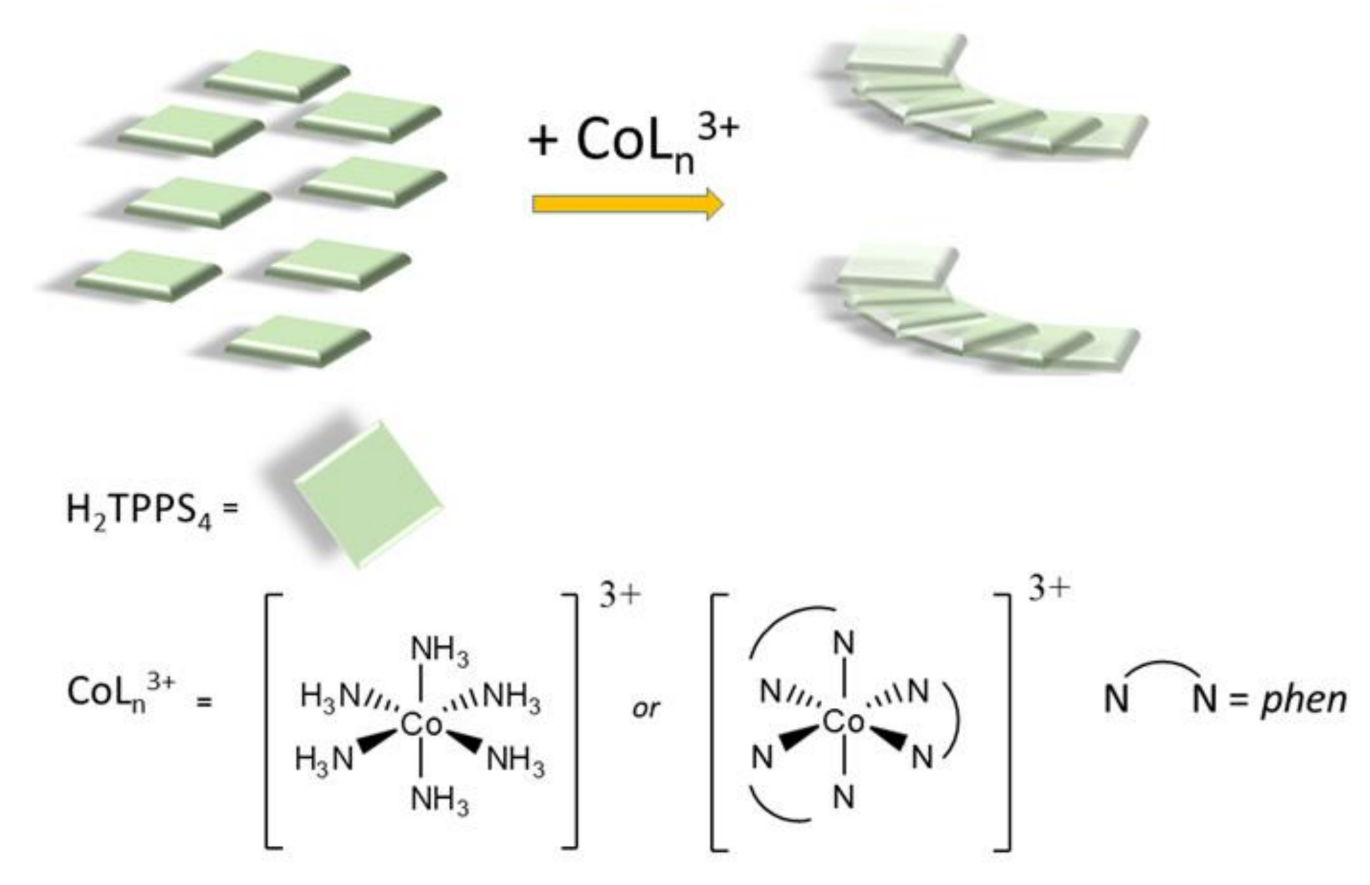
Pictorial sketch of the self-aggregation process of the diacid H_2_TPPS_4_ porphyrin into J-aggregates as induced by the cationic cobalt(III) complexes used in the present investigation.

Here we describe the outcome of a kinetic study on the supramolecular self-assembling process leading to J-aggregates of H_2_TPPS_4_, as induced by two cobalt(III) cationic complexes (Scheme 1). In these coordination compounds, cobalt(III) is a d^6^ low-spin metal ion, ensuring kinetic inertness under our experimental conditions. The ligands were selected to have hydrophobic (1,10-phenanthroline, phen) or hydrophilic (ammonia) character. We anticipate that (i) these two complex ions are efficient in triggering the formation of J-aggregates of H_2_TPPS_4_, at rather low concentration and at pH conditions that normally do not favor self-assembly of this porphyrin; (ii) the rate constants *k_c_* for the catalytic growth increase on increasing the complex concentration, with the amino complex being more effective than its *phen* analogue; (iii) the number of porphyrins, *m*, involved in the rate determining step are in the range 2–3, as already reported for the aggregation of H_2_TPPS_4_ under a variety of experimental conditions [77]; (iv) the intensity and shape of the J-bands of the aggregates in the final extinction spectra are strongly dependent on the cationic promoter, being much broader and less intense for the amine complexes, as consequence of different mechanisms for the electronic coupling between the chromophores; (v) symmetry breaking has been observed in these aggregated samples through the detection of dichroic signals, whose intensity decreases on increasing the aggregation rate. An inference on a common mechanism for the emergence of chirality in these self-assembled systems is at last proposed.

## 2. Results and Discussion

**Figure 1:**
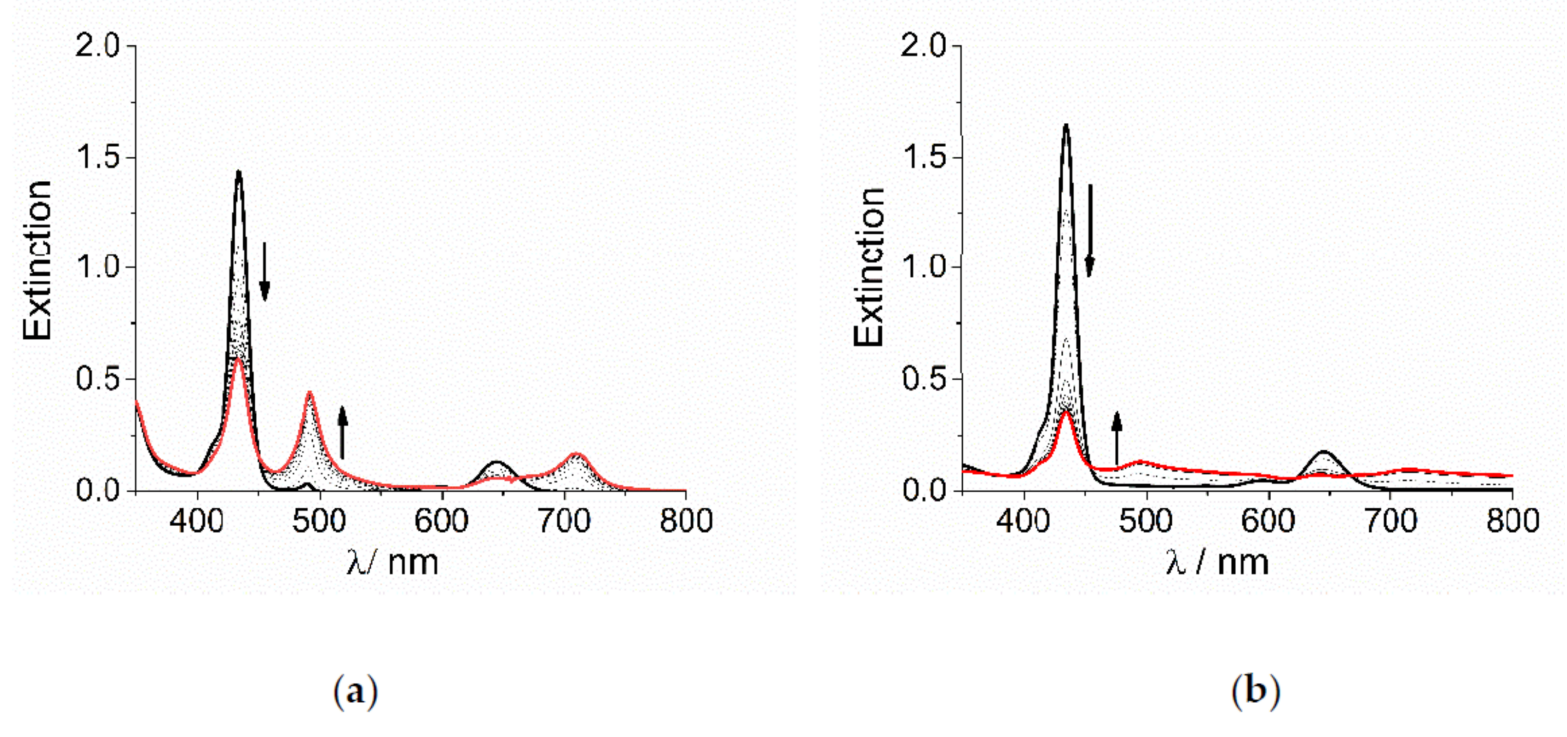
UV/Vis extinction spectral changes for the formation of J-aggregates of H_2_TPPS_4_ at pH = 2 upon addition of (**a**) [Co(phen)_3_]^3+^ and (**b**) [Co(NH_3_)_6_]^3+^ (Experimental conditions: [H_2_TPPS_4_] = 3 μM; [HCl] = 0.01 M, [CoL_n_^3+^] = 200 μM, T = 298 K, scanning time 450 s, total acquisition time 4000 s). The arrows indicate the decrease of the B-band at 434 nm and the matching increase of the J-band at 490 nm during the acquisition time.

TPPS_4_ porphyrin is a tetra-anionic species in aqueous solution at neutral pH. The central pyrrole-type nitrogen atoms have a pKa = 4.9 [78] and consequently they can be easily protonated at pH lower than 4, affording the dianionic diacid H_2_TPPS_4_. This species is able to self-aggregate at pH lower than 1 or alternatively, for intermediate pH values and ionic strength, *I*, higher than 100 *mM* [60,79]. At pH = 2 and in the absence of added salt, i.e., at low ionic strength, the diacid form is quite stable and aggregation occurs to a very low extent only after 24 h. When sub-millimolar concentration of the cobalt(III) complexes CoL_n_^3+^ are added to an acidified solution of H_2_TPPS_4_, UV/Vis extinction spectra display the typical changes for the conversion of the diprotonated monomeric porphyrin into its corresponding J-aggregate. The initial B-band of H_2_TPPS_4_ at 434 nm decreases in intensity, while a new J-band increases at about 490 nm, followed by changes in the Q-bands region (Figure 1). Anyway, the spectral changes observed for the two metal complexes are quite different in their overall appearance: for [Co(phen)_3_]^3+^ the J-band is rather sharp and with a moderate intensity (Figure 1a), while it appears very broad and weak for [Co(NH_3_)_6_]^3+^, with residual extinction spread over the entire investigated UV/Vis range (Figure 1b).

**Figure 2:**
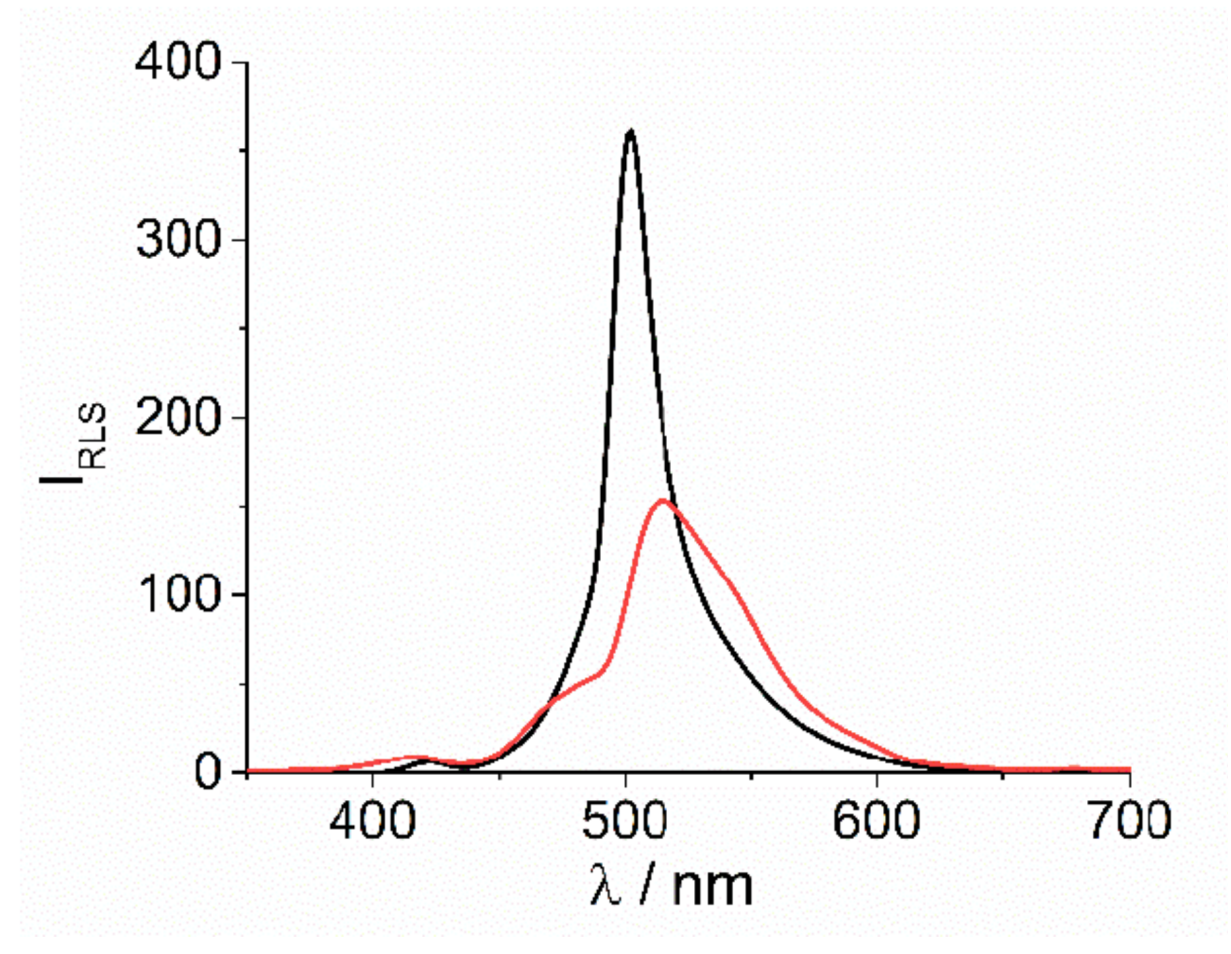
RLS profiles for the final J-aggregates of H_2_TPPS_4_ at pH = 2 upon the addition of [Co(phen)_3_]^3+^ (black solid line) and [Co(NH_3_)_6_]^3+^ (red solid line) (Experimental conditions: [H_2_TPPS_4_] = 3 μM; [HCl] = 0.01 M, [CoL_n_^3+^] = 200 μM, T = 298 K).

In both cases the presence of J-aggregates in solution is proved by resonant light scattering (RLS) technique. [12] Quite large intensity at resonance with the J-band at 500 and 512 nm, respectively for [Co(phen)_3_]^3+^ and [Co(NH_3_)_6_]^3+^, is well evident in the corresponding RLS spectra (Figure 2). According to the related extinction spectra, the much broader extinction feature observed for this latter sample corresponds to a lower and bathochromically shifted RLS peak. 

**Scheme 2:**
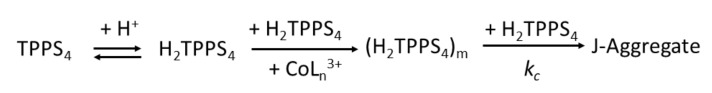
Mechanism showing the catalyzed pathway for the growth of J-aggregates from initial TPPS_4_ after protonation and addition of a cationic metal complex [CoL_n_]^3+^. In presence of the cationic promoter, the monomeric porphyrins form an oligomer containing *m* units in the rate-determining step. J-aggregates are eventually formed by further addition of monomers to the growing nano-assemblies.

The UV/Vis extinction versus time evolves following a typical sigmoidal form, in which after an initial lag-time an exponential growth dominates the remaining profile (Figure S1). The analysis of the kinetic traces at 490 nm, corresponding to the growth of the J-aggregates, has been performed applying a well-established model for these kind of supramolecular assembling processes [77,80]. Accordingly, the mechanism is based on two independent pathways: (i) an uncatalyzed one, that is controlled by a specific rate constant, *k*_0_; (ii) a catalyzed pathway, dominated by a rate constant *k_c_*, varying as a power *n* of time. This latter path is preceded by an early stage where the rate determining step is the formation of a porphyrin oligomer containing *m* monomers (see Scheme 2). This is the initial nucleus that triggers the following rapid growth of J-aggregates by incremental addition of monomeric units. A best fitting of the extinction data as function of time to the Equation (1) (see Experimental Section) allows to calculate the relevant kinetic parameters, whose values are collected in Table 1.

**Figure 3:**
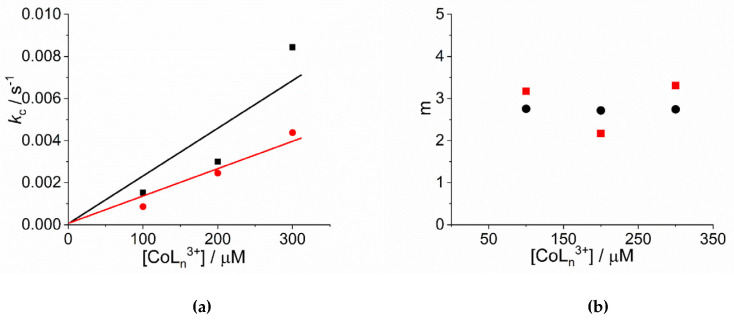

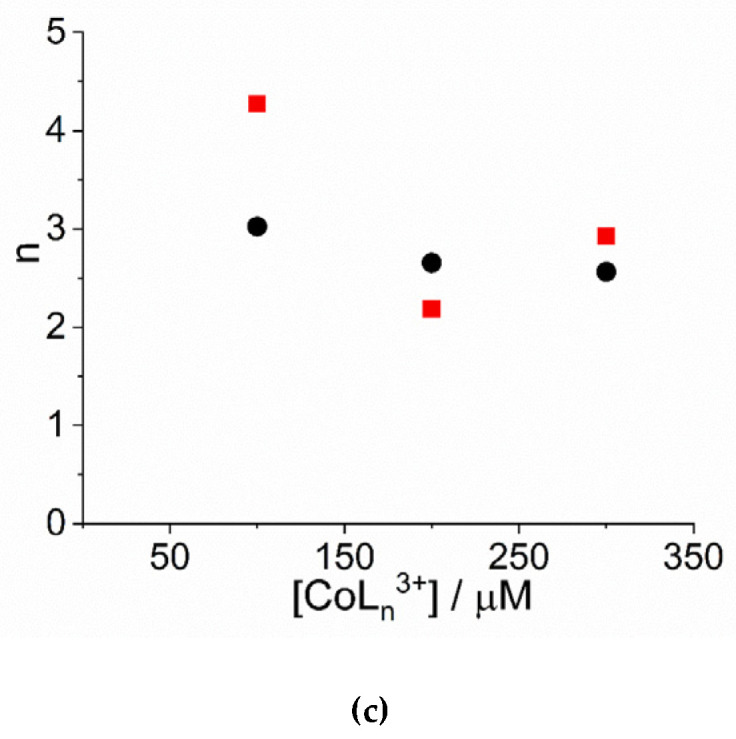
Plot of (**a**) the rate constants *k_c_* (s^−1^) and (**b**) the size of the critical nucleus, *m*, and (**c**) the time exponent *n* for the catalyzed growth of J-aggregates of H_2_TPPS_4_ at pH = 2 upon addition of [Co(phen)_3_]^3+^ (circles) and [Co(NH_3_)_6_]^3+^ (solid squares). (Experimental conditions: [H_2_TPPS_4_] = 3 µM; [HCl] = 0.01 M, T = 298 K). The solid lines in panel a represent the linear best-fits to the law *kc* = *kc’* × [CoL_n_^3+^].

An inspection of the data shows that the values of *k_c_* increase on increasing the concentration of cobalt(III) complexes. Figure 3a reports the linear best-fit of the rate data according to the rate law *k_c_* = *k_c_’* × [CoL_n_^3+^] ([Co(NH_3_)_6_]^3+^: *k_c_’* = (2.34 ± 0.44) × 10^−5^ µM^−1^, R^2^ = 0.9333; [Co(phen)_3_]^3+^: *k_c_’* = (1.31 ± 0.13) × 10^−5^ µM^−1^, R^2^ = 0.9803). The data have been treated also according to a second order rate law of the type: *k_c_* = *k_c_’’* × [CoL_n_^3+^] ([Co(NH_3_)_6_]^3+^: *k_c_’’* = (9.13 ± 0.66) × 10^−8^ s^−1^µM^−2^, R^2^ = 0.9681; [Co(phen)_3_]^3+^: *k_c_’’* = (5.05 ± 0.35) × 10^−8^ s^−1^µM^−2^, R^2^ = 0.9622) (Figure S2). In the case of [Co(NH_3_)_6_]^3+^ the deviation from a linear trend is more evident, thus favoring for a second order dependence. Unfortunately, due to the uncertainty in the kinetic data obtained for concentration of metal complex below 100 μM, an exact discrimination between the two different rate laws is not reliable. Concerning the size of the critical nucleus, the values of *m* are in the range 2 ÷ 3, so a dimer or trimer should be considered as the initial seeding aggregate (Figure 3b). These values are very similar to those reported for the self-assembling of H_2_TPPS_4_ under a variety of experimental conditions [65,77]. The values of the power exponent *n* range between 2 ÷ 4 (Figure 3c), again very similarly to other systems reported in literature [65,77]. As in other systems, the values for the rate constants of the uncatalyzed pathway, *k*_0_, contribute only to a small extent to the overall kinetic process and will not be further discussed. 

**Figure 4:**
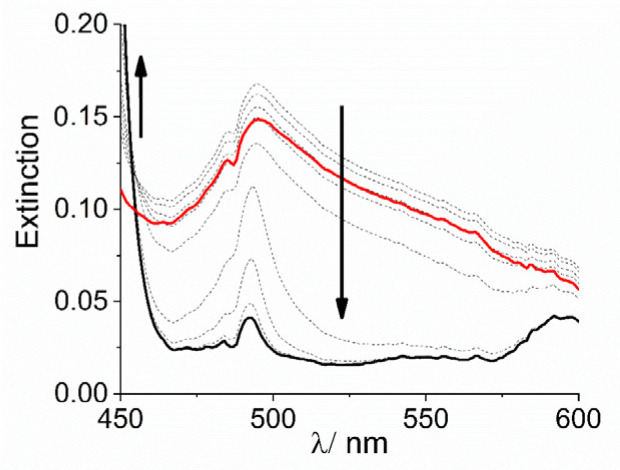
Expansion of UV/Vis extinction spectral changes in the J-band region for the disassembling of J-aggregates of H_2_TPPS_4_ stabilized with [Co(NH_3_)_6_]^3+^ at pH = 2 upon addition of PVS (Experimental conditions: [H_2_TPPS_4_] = 3 µM; [HCl] = 0.01 M, [Co(NH_3_)_6_] = 300 µM, [PVS] = 1 mM, T = 298 K, total acquisition time 1800 s). The arrows indicate the decrease of the broad extinction in the range 450–600 nm and the matching increase of the B-band at 434 nm during the acquisition time.

**Scheme 3:**
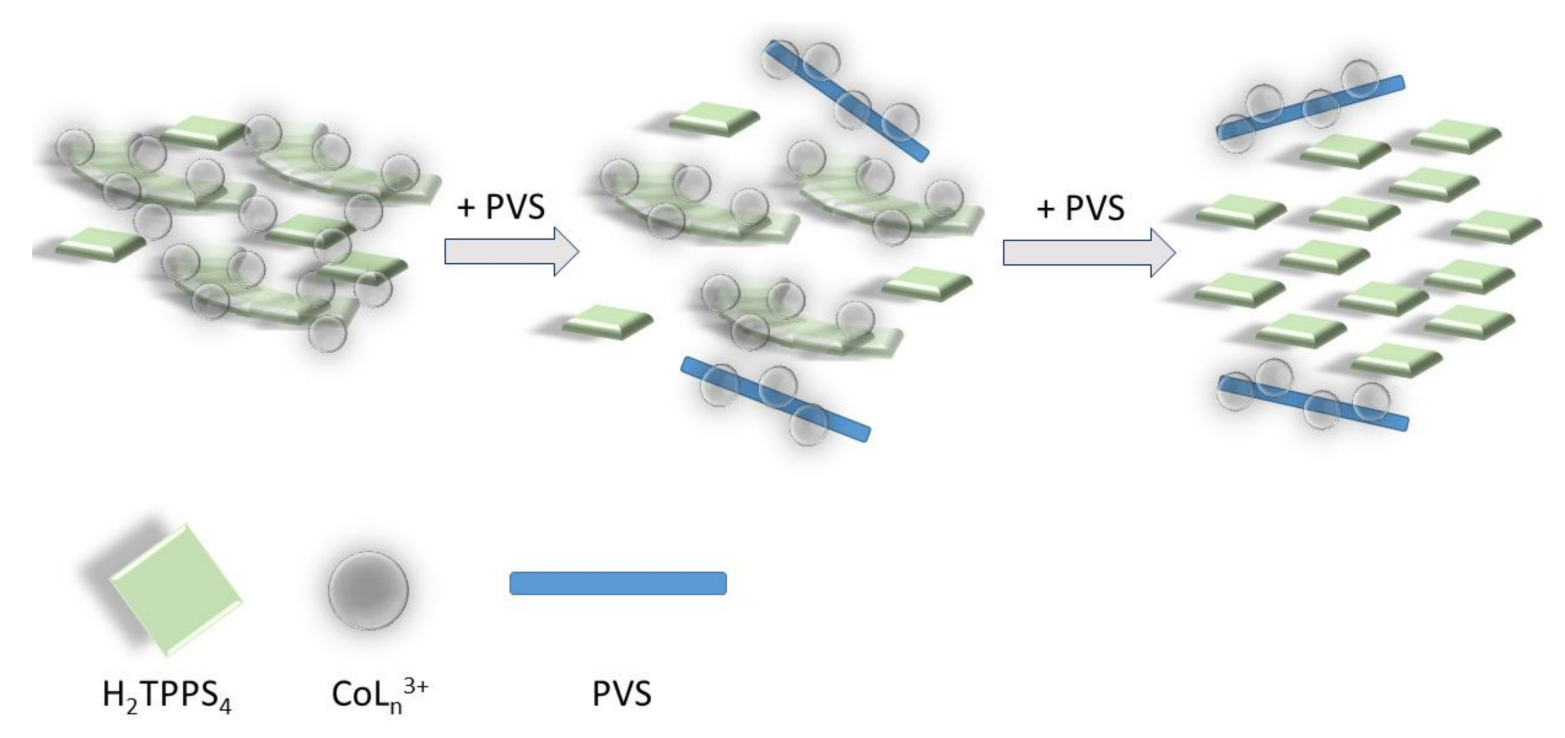
Pictorial sketch of the disassembling process mediated by the polyanionic PVS: in a first stage the polymer removes the cationic complex bridging monomeric porphyrin units in the J-aggregate mesoscopic structure. In this latter is evidenced the bridging role of cationic metal complexes with respect to H_2_TPPS_4_ nano-assemblies and monomeric units. In a second stage, the residual cationic complex is removed, and the J-aggregates disassemble into the constituent porphyrins.

Extinction and the intensity of RLS spectra on the final aggregated samples (Figure S3) increase on increasing the concentration of the cobalt complexes, i.e., on increasing the aggregation rates (Figure S4). Together with the kinetic data this evidence suggests that, whatever the concentration dependence is, the complex [Co(NH_3_)_6_]^3+^ is more efficient in accelerating the formation of these nano-assemblies. Apart from the ionic interactions that can be responsible for lowering the electrostatic barrier to the formation of initial oligomers between dianionic porphyrins, the observed difference between these cationic species could be due to various factors: (i) their sizes are different, with the ammonia affording a much more compact complex with respect to the bulkier 1,10-phenanthroline ligand; (ii) the nature of the interactions between the porphyrin and the complex ions are substantially different. In the case of the *phen* moiety, the planar aromatic surface of the ligand can lead to stacking interactions with the porphyrin ring or with the phenyl substituent groups. This fact could determine a more rigid and intimate contact between the two species. In the case of ammonia, hydrogen bonding between N-H groups and sulfonate ionized group can occur, leading to stabilization of the nano-aggregate and to potential bridging among these latter and even monomeric H_2_TPPS_4_ units. This hypothesis is not unprecedented since previous investigations showed similar effect played by a tetracationic amine (spermine) in driving a complex mesoscopic structure were the polycation acts as electrostatic “glue” in solution, becoming part of the nano-aggregates and their mesoscopic architecture [16,67]. To support this idea, we performed some experiments to check the stability of the system upon addition of a scavenger of cationic species. Poly(vinyl-sulfonate) (PVS) is a highly charged anionic polyelectrolyte and its strong electrostatic field is effective in condensing oppositely charged cations in solutions [81]. When a solution of this polymer is added to a preformed H_2_TPPS_4_ J-aggregate, under the experimental conditions used in our study, almost no change occurs if [Co(phen)_3_]^3+^ is used as promoter species and the solution remains unaltered for at least 24 h. On the contrary, when J-aggregates have been triggered by [Co(NH_3_)_6_]^3+^, addition of PVS leads to an initial decrease and substantial sharpening of the broad J-band, followed by its almost complete disappearance (Figure 4), paralleled by the matching increase of the B-band at 434 nm, due to the free monomeric H_2_TPPS_4_ (Figure S5). These experimental findings are in agreement with a mesoscopic structure stabilized by an interplay of hydrogen bonding and electrostatic interactions among porphyrin monomers and nano-J-aggregates mediated by the amino-complex (Scheme 3). Initially PVS acts as scavenger only for externally bound and bridging cationic complexes, favoring the release of porphyrin units and intact nano-assemblies. These latter species are responsible for the sharp absorption feature at 490 nm [67]. Eventually, the extraction of intimately bound cationic complexes from the nano-assemblies determines their gradual disassembling process with concomitant release of free monomeric units.

The hypothesis of a mesoscopic architecture as that above depicted is further corroborated by the difference of the extinction spectra in the two cases. As already outlined describing Figure 1, the appearance of the extinction feature for the final J-aggregates is deeply dissimilar when these species form in presence of the two cationic complexes. When considering [Co(phen)_3_]^3+^, the J-band displays a full-width at half-maximum, FWHM, in the range 860 cm^−1^. Even if a correction for the light scattering contribution should be applied, these values are already larger than those usually found for J-aggregates prepared at lower pH, where they exhibit a nanotubular morphology. In these latter kind of nanostructures, the tight packing of the chromophores and the electronic coupling can be described according to the Frenkel exciton model [82]. The corresponding observed FWHM in the electronic spectra is related to the coherence length of the exciton or the spectroscopic aggregation number N, according to the equation FWHM ∝ N^−1/2^. As consequence of the considerable coherence length in the nanotubes, the observed linewidth is usually much more narrow (244 cm^−1^) with respect to the bandwidth of the B-band of the parent diacid porphyrin (875 cm^−1^) [83]. In the case of the complex [Co(NH_3_)_6_]^3+^, the envelop of the J-band is very broad and therefore the model for the electronic coupling should be different. Actually, these UV/Vis extinction spectra are more like those exhibited by colloidal metal particles and can be modeled through a dipolar coupling mechanism. Previous investigation on H_2_TPPS_4_ J-aggregates induced by spermine [16] showed that their electronic spectra are dominated by this type of coupling and are very similar to the present case. The observed difference in the behavior of rate constants *k_c_* vs. [CoL_n_^3+^], i.e., a linear dependence for the *phen* complex, while a non-linear or second order dependence for the amino-complex, seems to further support the difference in their mesoscopic structure. 

**Figure 5:**
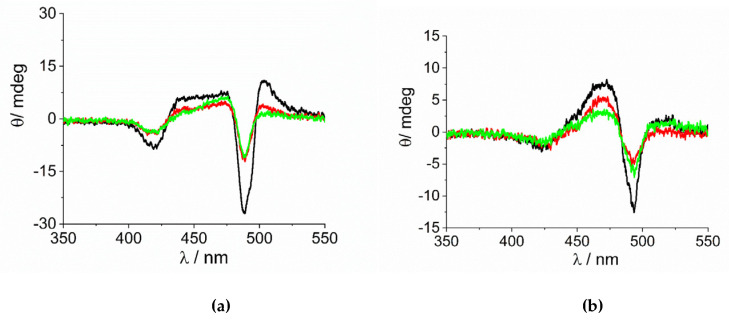
CD spectra of the samples at the end of the aggregation process for the experiments at variable concentration of (**a**) [Co(phen)_3_]^3+^ and (**b**) [Co(NH_3_)_6_]^3+^(Experimental conditions: [H_2_TPPS_4_] = 3 µM; [HCl] = 0.01 M, [CoL_n_^3+^]= 100 µM (black line), 200 µM (red line), 300 µM (green line), T = 298 K).

**Figure 6:**
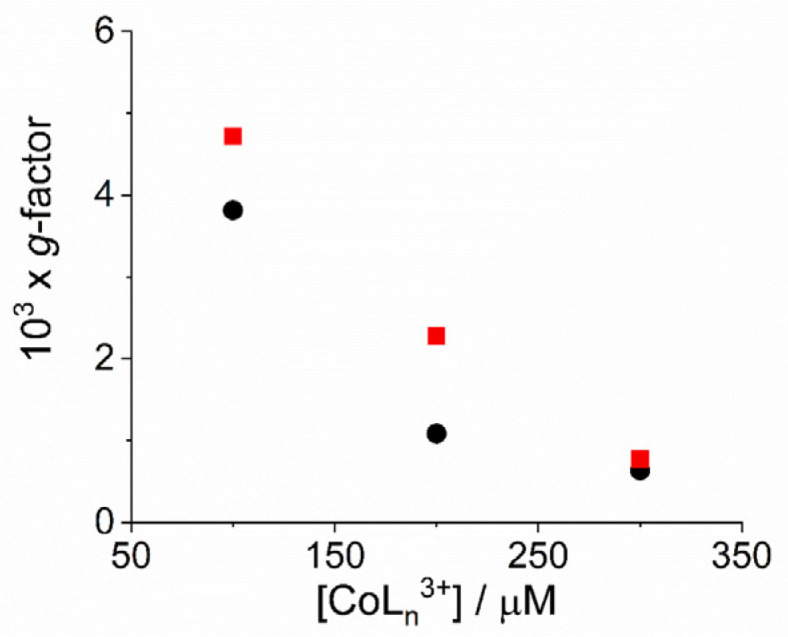
Plot of dissymmetry *g*-factor for the fully aggregated samples as function of the concentration of the complex metal ions [Co(phen)_3_]^3+^ (circles) and [Co(NH_3_)_6_]^3+^ (solid squares). (Experimental conditions: [H_2_TPPS_4_] = 3 µM; [HCl] = 0.01 M, T = 298 K).

**Figure 7:**
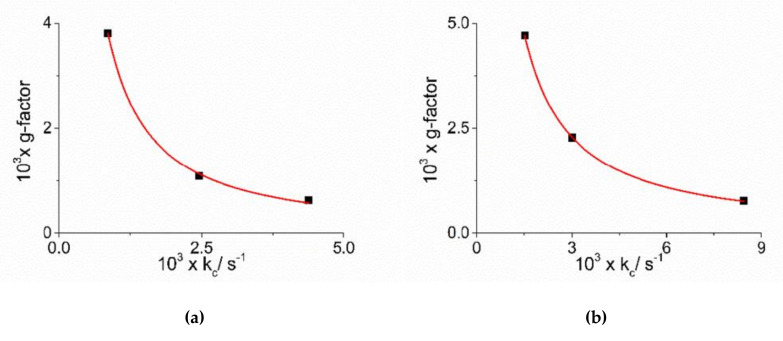
Plot of dissymmetry *g*-factor versus the catalytic rate constants *k_c_* for J-aggregates obtained in the presence of the complex metal ions [Co(phen)_3_]^3+^ (**a**) and [Co(NH_3_)_6_]^3+^ (**b**). The red line represents the non-linear fit of data to the equations: g = α × *k_c_*^β^ ([Co(phen)_3_]^3+^: α = (1.07 ± 0.37) × 10^−6^, β = −(1.16 ± 0.05), R^2^ = 0.99824; [Co(NH_3_)_6_]^3+^: α = (4.73 ± 0.34) × 10^−6^, β = −(1.06 ± 0.01), R^2^ = 0.99989).

As already reported in literature, J-aggregates of the H_2_TPPS_4_ porphyrin could exhibit spontaneous symmetry breaking [62,77,84]. Actually, circular dichroism (CD) spectroscopy is a very informative technique about the occurrence of chirality in a molecular or aggregated system. In porphyrin J-aggregates Cotton effect is induced in resonance with the corresponding electronic absorption, giving very distinctive exciton split bands. This issue is particularly intriguing since the starting neutral porphyrin is not chiral, while its nanoassemblies could exist as enantiomorphous pairs. Considering the stochastic nature of the aggregation process, a distribution of nano-aggregates having different sizes is the most probable scenario in solution, thus affording a quasi-racemic system and evidencing an almost perfect balance in their chiroptical response. The unbalance in the population leads therefore to the appearance of exciton couplets in the corresponding CD spectra. Figure 5 shows that all the samples containing J-aggregates induced by the investigated metal complexes exhibit spontaneous symmetry breaking, as proved by bisegnated positive CD bands centered at the J- and H-bands of the nano-aggregates. Both set of CD spectra are affected by differential circular light scattering that slightly modifies the envelop of the CD features, especially in the case of the amino-complex, where the longer wavelength component of the J-couplet is consistently reduced with respect to the *phen* complex. The dissymmetry g-factor (Δε/ε) is indicative of the quality of the CD oscillator and the values for both systems decrease on increasing [CoL_n_^3+^] (Figure 6). Since the rate constants *k_c_* for the catalyzed growth of these J-aggregates also increase with the concentration of the metal complexes, this implies that g-factor decreases on increasing the aggregation rates with an inverse dependence of the type *g* = *constant*/*k_c_* (Figure 7). This experimental finding is similar to what observed in other analogous J-aggregates [84]. On increasing the rate of the self-assembly formation, the potential occurrence of defects or meso-forms of the nano-aggregates could become more relevant, decreasing or nulling the CD signal.

## 3. Materials and Methods

### 3.1. Materials 

The sodium salt of 5, 10, 15, 20-tetrakis(4-sulfonatophenyl)porphyrin (TPPS4) was purchased from Aldrich (Milan, Italy). Hexamminecobalt(III) chloride [85] and tris(1,10-phenanthroline)cobalt(III) chloride [86] were synthesized according to the literature. Hydrochloric acid (Fluka, Milan, Italy) was of the highest commercial grade available and was used as received without further purification. Poly(vinylsulfonate) sodium salt was obtained from Aldrich (Milan, Italy) as aqueous solution (30%). All the aqueous solutions were prepared in high-purity doubly distilled water (HPLC grade, Fluka, Milan, Italy). A stock solution of porphyrin was freshly prepared and stored in the dark. The concentration used in the experiments was calculated by UV/Vis absorption spectroscopy using the molar extinction coefficients at the B-band (TPPS_4_: 5.33 × 10^5^ M^−1^cm^−1^, λ = 414 nm). Polyelectrolyte concentration, expressed as moles of sulfonate units per liter, was obtained by weighing. To avoid or minimize dust contamination, special care was taken by filtering all the stock solutions through 0.22 mm Millipore filters.

### 3.2. Methods 

UV/Vis extinction spectra were acquired on an Agilent 8453 diode array spectrophotometer. An UV filter (Hoya glass type UV-34, cut-off: 340 nm) was always set in the light path between the lamp and the measurement cell to avoid any photo-damage of porphyrin samples, especially during kinetic experiments. CD spectra were measured on a Jasco J-710 spectropolarimeter. The dissymmetry g-factor is calculated from the experimental CD spectra and the corresponding extinction spectra, using the formula g = Δε/ε = ΔA/A, where ΔA = Θ/32980 (ΔA is in absorbance units and Θ is the ellipticity in mdeg). Resonance light scattering (RLS) spectra were acquired on a Jasco FP-750 spectrofluorimeter, adopting a synchronous scanning protocol of both excitation and emission monochromators and a right angle geometry [12]. 

Kinetic runs were executed by acquiring spectra from cells placed in the thermostatic sample holder of the spectrophotometer (298 K). All the reactions were started by adding a known aliquot of an aqueous solution of the metal complexes to a pre-acidified solution of TPPS_4_ (3 μM) (HCl, pH = 2). To ensure an adequate mixing, solutions were inverted at least three times. The kinetic parameters were obtained from extinction versus time. The experimental extinction data collected at 490 nm were analyzed by a non-linear least square fit to the equation: Ext_t_ = Ext_∞_ + (Ext_0_ − Ext_∞_) (1 + (*m* − 1){ *k*_0_t + (*n* + 1)^−1^ (*k_c_* t)*^n^*^+1^})^−1/(*m*−1)^ (Equation (1)), where Ext_0_, Ext_∞_, *k*_0_, *k_c_*, m and n are the parameters to be optimized. 

## 4. Concluding Remarks

J-aggregates of water soluble sulfonated porphyrins are intriguing supramolecular systems, since they can afford a large variety of structural motifs spanning from nano up to micrometer scale, depending on the selected experimental conditions and mixing protocols. Their chiroptical properties can be finely tuned by a proper choice of chiral templating reagents, even if spontaneous symmetry breaking is a commonly observed phenomenon. In this investigation we contribute to gain some more knowledge on the role of species that can promote porphyrin self-assembly formation and stabilization, even under not-aggregating conditions. Kinetic investigations offer the advantage to acquire information on the mechanism of growth and to learn more on the factors controlling the aggregation. Metal complexes are an invaluable tool to access hybrid organic-inorganic nano-systems, since they can introduce redox activity, photochemical and catalytic properties, so enlarging their field of potential applications.

## Figures and Tables

**Table 1:** Relevant kinetic parameters for the J-aggregation of H_2_TPPS_4_ (*k*_0_, *k_c_*, *m* and *n*) as function of [CoL_n_^3+^] (pH = 2, T = 298 K).

	10^4^ × *k*_0_/ s^−1^	10^3^ × *k_c_*/ s^−1^	*m*	*n*
		[Co(NH_3_)_6_]^3+^		
100	0.65 ± 0.16	1.52 ± 0.01	3.2 ± 0.1	4.3 ± 0.2
200	-	3.00 ± 0.01	2.2 ± 0.1	2.2 ± 0.1
300	3.27 ± 0.98	8.44 ± 0.04	3.3 ± 0.1	2.9 ± 0.1
		[Co(*phen*)_3_]^3+^		
100	2.85 ± 0.17	0.863 ± 0.013	2.8 ± 0.1	3.0 ± 0.2
200	5.41 ± 0.27	2.46 ± 0.01	2.7 ± 0.1	2.7 ± 0.1
300	5.17 ± 0.37	4.38 ± 0.01	2.7 ± 0.1	2.6 ± 0.1

from non-linear fit of extinction data to eq. 1 at 490 nm. In most of the examined cases, the term *k*_0_ makes a statistically irrelevant contribution to the overall process.

## Data Availability

Please refer to suggested Data Availability Statements in section “MDPI Research Data Policies” at https://www.mdpi.com/ethics.

## Supplementary Materials

The following are available online at https://www.mdpi.com/1422-0067/22/1/39/s1, Figure S1: Typical extinction time trace; Figure S2: Plot of the rate constants *k_c_* (s^−1^) vs. metal complexes concentration; Figure S3: Plot of extinction and RLS intensity vs. metal complexes concentration; Figure S4: Plot of RLS intensity vs. the rate constants *k_c_* (s^−1^); Figure S5: UV/Vis spectral changes for PVS interacting with [Co(NH_3_)_6_]^3+^ J-aggregates
