# Supplementary material for: Role of Cobalt(III) Cationic Complexes in the Self-Assembling Process of a Water Soluble Porphyrin
Source: Int J Mol Sci. 2020 Dec 22;22(1):39. doi: 10.3390/ijms22010039 (PMC7792976; doi:10.3390/ijms22010039)
Supplement: Supplementary file 1 [file ijms-22-00039-s001.pdf]

Supporting Information

for

Role of Cobalt(III) Cationic Complexes in the Self-assembling Process of a Water Soluble Porphyrin

[1] Dipartimento di Scienze Chimiche, Biologiche, Farmaceutiche ed Ambientali, University of Messina and C.I.R.C.M.S.B V.le F. Stagno D'Alcontres, 31 - 98166 Messina, Italy E.mail nadiamanganaro87@gmail.com (N.M.); anromeo@unime.it (A.R.); lmonsu@unime.it (L.M.S.)
[2] CNR - ISMN Istituto per lo Studio dei Materiali Nanostrutturati c/o Dipartimento di Scienze Chimiche, Biologiche, Farmaceutiche ed Ambientali, University of Messina, V.le F. Stagno D'Alcontres, 31 - 98166 Messina, Italy roberto.zagami@ismn.cnr.it (R.Z.); mariachiara.trapani@cnr.it (M.T.); maria.castriciano@cnr.it (M.C.)
* Correspondence: lmonsu@unime.it; Tel.: +39 090 6765711

Content:

Fig. S1. Typical extinction time trace p. 2

Fig. S2. Plot of the rate constants k_c (s^{-1}) *vs* metal complexes concentration p.3

Fig. S3. Plot of extinction and RLS intensity *vs* metal complexes concentration p.4

Fig. S4. Plot of RLS intensity vs the rate constants k_c (s^{-1}) p.5

Fig. S5. UV/Vis spectral changes for PVS interacting with $[Co(NH_3)_6]^{3+}$ J-aggregates p.6

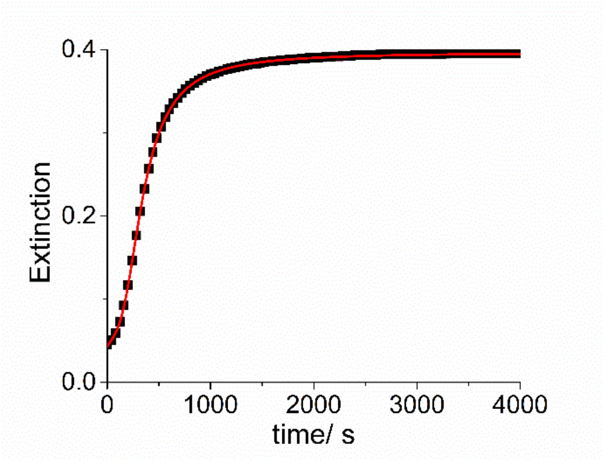

Figure S1. Typical extinction time trace for the formation of J-aggregates of H_2TPPS_4 at pH = 2 upon addition of $[Co(phen)_3]^{3+}$ (Experimental conditions: $[H_2TPPS_4]$ = 3 μM; [HCl] = 0.01 M, $[Co(phen)_3^{3+}]$ = 200 μM, T = 298 K). The solid line represents the best-fitted curve to the experimental data according eq. 1.

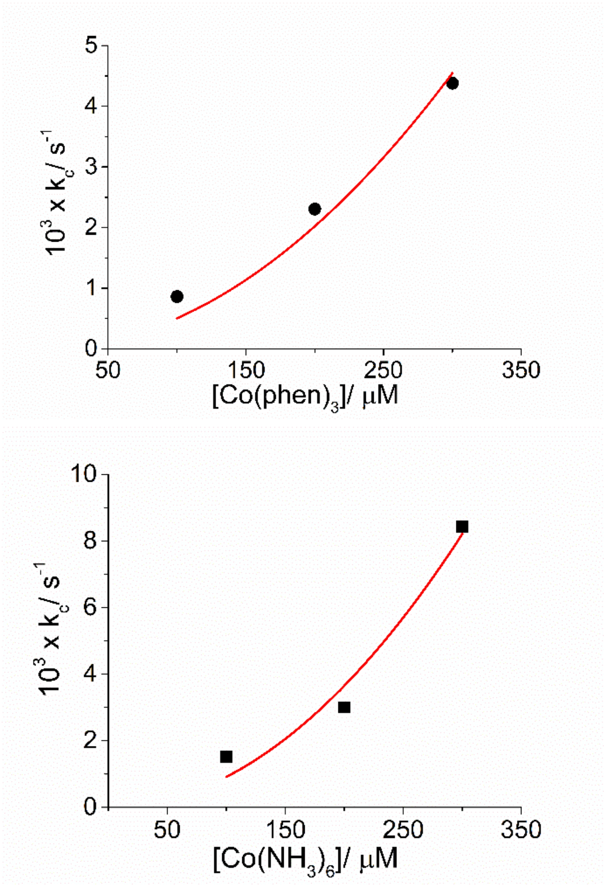

Figure S2. Plot of the rate constants k_c (s^{-1}) for the catalyzed growth of J-aggregates of H_2TPPS_4 at pH = 2 upon addition of $[Co(phen)_3]^{3+}$ (circles) and $[Co(NH_3)_6]^{3+}$ (solid squares). (Experimental conditions: $[H_2TPPS_4]$ = 3 μM; [HCl] = 0.01 M, T = 298 K). The solid lines represent the non-linear best-fits to the law $kc = kc' \times [CoL_n^{3+}]^2$ ($[Co(NH_3)_6]^{3+}$: k_c'' = (9.13 ± 0.66) × 10^{-8} $s^{-1}\mu M^{-2}$, R^2 = 0.9681; $[Co(phen)_3]^{3+}$: k_c'' = (5.05 ± 0.35) × 10^{-8} $s^{-1}\mu M^{-2}$, R^2 = 0.9622).

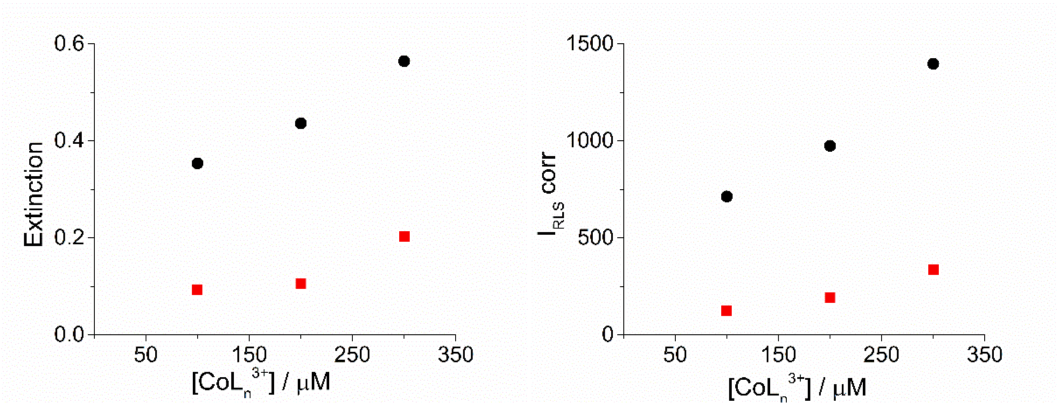

(a) **(b)**

Figure S3. Plot of (a) the extinction values and (b) the RLS intensity at maxima corrected for the extinction of the samples as function of $[Co(phen)_3]^{3+}$ (circles) and $[Co(NH_3)_6]^{3+}$ (solid squares) (Experimental conditions: $[H_2TPPS_4]$ = 3 μM; [HCl] = 0.01 M, T = 298 K).

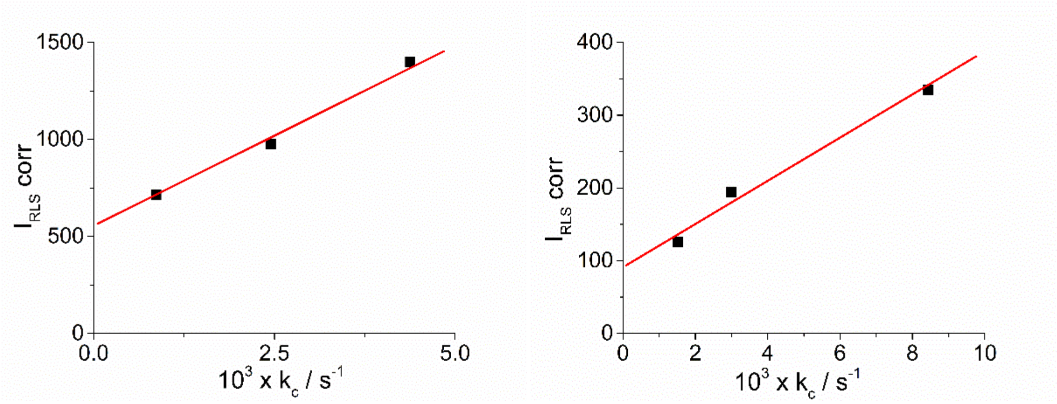

(a) (b)

Figure S4. Plot of the RLS intensity at maxima corrected for the extinction of the samples as function of the corresponding rate constants k_c for the catalyzed growth of J-aggregates of H_2TPPS_4 at pH = 2 upon addition of a) $[Co(phen)_3]^{3+}$ and b) $[Co(NH_3)_6]^{3+}$. (Experimental conditions: $[H_2TPPS_4]$ = 3 μM; [HCl] = 0.01 M, $[CoL_n^{3+}]$= 100 μM, 200 μM an 300 μM, T = 298 K). The solid lines represent the linear best-fits to the equations: a) I_{RLS}^{corr} = (527 ± 47) + (1.95 ± 0.16)× 10^5 × $[Co(phen)_3]$ (R^2 = 0.9934); b) I_{RLS}^{corr} = (93 ± 18) + (2.90 ± 0.35)× 10^4 × $[Co(NH_3)_6]$.

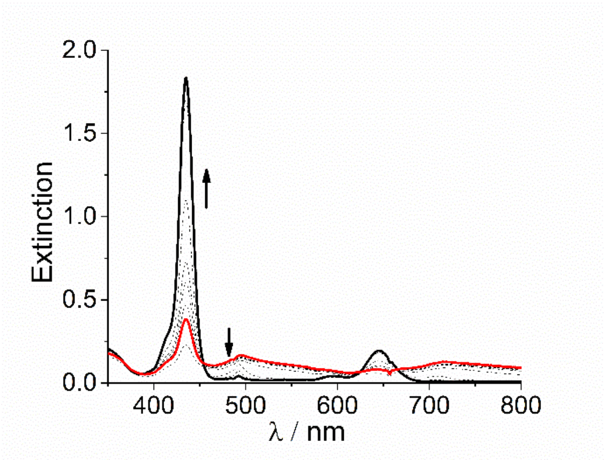

Figure S5. UV/Vis extinction spectral changes for the disassembling of J-aggregates of H_2TPPS_4 stabilized with $[Co(NH_3)_6]^{3+}$ at pH = 2 upon addition of PVS (Experimental conditions: $[H_2TPPS_4]$ = 3 μM; [HCl] = 0.01 M, $[Co(NH_3)_6]$ = 300 μM, [PVS] = 1 mM, T = 298 K, total scanning time 1800 s).
